# Supplementary material for: Are carbon emissions trading and green financial instruments synergistic? -Comprehensive quantitative research based on content analysis
Source: PLoS One. 2024 Mar 7;19(3):e0298601. doi: 10.1371/journal.pone.0298601 (PMC10919601; doi:10.1371/journal.pone.0298601)
Supplement: S1 Data — (ZIP) [file pone.0298601.s001.zip › Data/地级市Z_F工作报告.zip!/╜¡╦╒/╬▐╬2╩╨/└┤╫╘╣1⁄2╓┌║┼í╛┬φ┐╦╩2╛▌═°í┐_2011.docx]

各位代表：

现在，我代表无锡市人民政府，向大会报告工作，请予审议，并请市政协委员和其他列席人员提出意见。

2010年工作回顾及“十一五”发展成就

2010年是“十一五”最后一年，在中共无锡市委领导下，市政府紧紧依靠全市人民，着力打造“生态城、高科技产业城、旅游和现代服务城、宜居城”，经济社会实现平稳较好发展。预计，实现地区生产总值5750亿元，同比增长13%左右；财政总收入1579.9亿元，同口径增长20.7%，其中一般预算收入511.9亿元，增长23.1%；城镇居民人均可支配收入增长11.5%左右，农民人均纯收入增长12%左右；城镇登记失业率控制在2.6%以内，各项事业和各项工作取得新的显著进展，圆满完成了“十一五”发展任务。

（一）完善增长机制，国民经济持续较快发展

消费需求旺盛。适应群众生活多样化、个性化需要，积极发挥消费对经济增长的拉动作用，促进消费各项政策有效落实。科学布局商业网点，商贸流通市场体系日趋完善。市区10家农贸市场完成标准化升级改造，全市涉农行政村为农服务社实现全覆盖。全年完成社会消费品零售总额1809.1亿元，增长18.6%；市场成交总额3000亿元，增长20%。全力保障市场供应，强化价格监管，向低收入群体发放物价补贴2369万元。居民消费价格指数103.4，物价涨幅低于全省平均水平。投资结构改善。拓宽社会投资领域，激发民间投资活力，稳定政府投资规模。海太半导体封装测试、长三角金属物流园等29个市级重点项目建成。全社会固定资产投资完成2985.7亿元，增长25.1%；其中民间投资占比达53.1%，服务业投资占比达58.9%。开放型经济回升向好。利用外资结构趋优，引进独立研发中心、地区总部、金融机构25家，到位注册外资33亿美元。对外贸易攀升，进出口总额612.5亿美元，增长39.4%；其中出口总额362.9亿美元，增长39.5%。“走出去”步伐加快，新批境外投资项目59个。全市国际友城增加到36个，荣获国际友好城市交流合作奖。体制机制活力增强。加快存量国有资产整合，全面完成国有企业清产核资，国有资本在关键领域的集中度增强，市属国有企业资产规模达2064亿元。加快建立集投资、融资、担保等功能于一体的企业服务体系，无锡产权交易所中小企业产权交易及融资服务中心、无锡市股权登记托管服务中心挂牌营业。推动各类企业直接上市融资，新增上市企业17家，首发融资122亿元。

（二）推动产业升级，经济结构调整取得重要进展

创新资源集聚效应凸显。积极开展“千人计划促进年”活动，引进海外领军型创新创业人才653名，6人入选国家“千人计划”。加快区域创新体系建设，累计建成省级以上高技术研究实验室5个、科技公共服务平台65个、工程技术研究中心240个。长电科技、美新半导体入选国家第四批创新型试点企业。预计，全社会研发费用占地区生产总值比重达2.5%。成功举办上海世博会“科技创新与城市未来”主题论坛、中国（无锡）海归创新创业峰会。制造业水平提升。新认定的高新技术企业增加到640多家，新增国家重点新产品36项，省级高新技术产品总数达624个。预计，高新技术产业增加值占规模以上工业增加值比重提高到45.7%。新增驰名商标12件，营业收入超百亿元企业增加到22家，法尔胜集团、远东集团被授予第三届市长质量奖。无锡入围国家知识产权工作示范城市和首批国家商标战略实施示范城市。物联网产业快速发展，预计实现销售收入360亿元，八大战略性新兴产业营业收入增长29%。市区107家工业企业完成整体搬迁或关停整合，其中锡钢集团等12家企业退城出市，市区工业布局调整工作基本完成。服务业发展加快。预计，服务业增加值占地区生产总值比重提高到42.6%。借助上海世博会效应，加快旅游目的地建设，全年接待入境旅游者86.5万人次，预计旅游总收入730亿元。创意设计、动漫游戏等产业快速发展，预计文化产业增加值占地区生产总值比重达6%。金融运行态势良好，本外币各项存、贷款余额分别为8827.2亿元和6487.1亿元，创业投资基金规模突破103亿元。服务外包强劲攀升，执行金额25.3亿美元，产业规模继续保持全国示范城市前三位。积极贯彻国家房地产市场调控政策，努力保持市场稳定，全市商品房销售面积1010.2万平方米。农业现代化水平提高。加强农业标准化建设，推动农业生态化发展，新增高效农业面积5100多公顷，拥有市级以上名牌农产品116个。大力推动生物农业集聚发展，生物农业产值超过19亿元。

（三）统筹城乡建设，城市化现代化水平显著提升

城市综合功能进一步完善。以公共设施建设和功能开发为切入，不断强化新城和重点片区集聚引领效应。市民中心、太湖国际博览中心一期投入运行，万达商业广场等城市综合体建成营业。推进古街、古镇、古村保护建设，鸿山遗址公园入选第一批国家考古遗址公园，清名桥历史文化街区荣膺十大“中国历史文化名街”称号，长泾镇、荡口镇、礼舍村入选第五批中国历史文化名镇、名村。基础设施承载力进一步增强。地铁1号线24个站点全面开建，2号线启动建设。沪宁城际高铁无锡站及惠山站、新区站正式启用，京沪高铁无锡站完成主体结构。苏南硕放机场集团正式成立，无锡机场全年进出港旅客253.5万人次、货邮吞吐量5.7万吨。苏南运河无锡段航道升级整治工程基本完成。全市港口货物吞吐量突破2亿吨，无锡江阴港集装箱吞吐量101.1万标箱。优化城乡路网结构，提升道路通行能力，锡张高速公路建成通车，市区新建及改拓建城市道路289千米，全市新改建农村公路241千米，新改建农村危旧桥梁272座。扩大公交服务区域，新增公交线路22条，市区公交日均运量达到115万人次。城乡管理进一步加强。推进城市管理重心下移，社区（村）全面设立城管站。强化综合整治，市区包装出新道路14条，市容环境秩序明显改善。沪宁城际高铁无锡段沿线环境整治获省级评比一等奖。农村新五件实事全部完成。万顷良田建设全面推进，完成土地整理4904.6公顷。全市386个自然村完成村庄环境综合整治，495个自然村居民并入城镇或农村新型社区。新增现代化新农村建设示范镇6个、示范村70个。

（四）构建生态文明，环境质量持续改善

太湖治理深入推进。提前两年基本完成国家治太总体方案确定的重点工程。梅梁湖、月亮湾生态清淤完成289万立方米，建成环太湖生态防护林1667公顷，完成梁鸿湿地等入湖河道湿地恢复工程。城北污水处理厂四期、太湖新城污水处理厂中水回用工程投运，累计建成排水达标区3588块，全市污水管网总长度增加到7612千米，68座污水处理厂全部达到一级A排放标准，日污水处理能力超200万吨，预计城镇污水集中处理率超过86.5%。太湖无锡水域水质改善，主要饮用水源地水质达标率100%，12个国家考核断面水质达标率100%，实现安全度夏，确保了城乡用水安全。节能减排完成良好。135个减排工程全面完成，关闭重污染企业123家。对化工、冶金、琉璃瓦和热电行业实施整治，全年关停企业389家，整改221家。加强节能监督和能源计量，严格项目能效评估，有效遏制高耗能项目建设。积极发展循环经济，306家企业完成清洁生产审核验收。生态创建成效显著。环城古运河风貌带综合整治圆满完成，锡惠名胜区入口公园、金匮公园建成开放，蠡湖景区东扩有序推进。锡东生活垃圾焚烧厂启用，桃花山垃圾填埋厂扩建一期工程完成。“军地携手保护太湖”、“军民同心共建美好家园”活动成效明显。全年造林绿化4367公顷，预计全市林木覆盖率达24.5%，全市新增城市公园绿地面积271万平方米，其中市区新增175万平方米。累计建成国家级环境优美乡镇45个，市级以上生态村386个，市级以上绿色社区344个。预计环境质量综合指数90。我市顺利通过生态市国家级考核验收，无锡市、江阴市入选全国生态文明建设试点城市。

（五）加强社会建设，公共服务能力有效增强

教育事业优先发展。预计全市教育投入增长22.4%以上，其中市本级增长25.2%。投入7.8亿元完成67所义务教育相对薄弱学校改造，农村义务教育学校装备条件全部达到省颁Ⅱ类标准。无锡城市职业技术学院新校区建成。北大软微学院无锡产学研合作教育基地等一批高教资源落户无锡。卫生改革发展不断深入。加快医疗卫生资源优化整合，推进城乡社区卫生服务中心标准化建设，基层医疗卫生机构全面启动实施国家基本药物制度，药品平均价格较市场零售价下降42%以上。市二院综合病房大楼建成投用。文明程度稳步提升。创建全国文明城市取得新进展，市民文明素质进一步提高。市、市（县）区两级文化馆、图书馆、博物馆实行全年无休开放服务，中国乡镇企业博物馆、中国民乐博物馆、无锡书画博物馆等建成开放。完成国有文艺院团事转企改革，组建成立市演艺集团。新增公共体育设施面积71.7万平方米，无锡运动员参加亚运会、亚残会、省运会取得好成绩。社会管理有效加强。全面推开社区扁平化管理改革，累计建成城市社区事务工作站554家、农村社区服务中心613家。重点开展世博食品安全保障、放心豆制品工程等专项行动，无锡成为全国首批“肉菜追溯体系”试点城市，食品安全形势基本平稳。人口和计划生育公共服务进一步强化，全国人口普查工作初见成效。全国经济普查任务圆满完成。气象公共服务保障水平提高。全市未发生重特大安全事故，各类生产安全事故起数和死亡人数连续九年实现“双下降”。无锡被评为全国人民防空先进城市。落实社会矛盾化解、社会管理创新、公正廉洁执法三项重点工作，健全社会治安防控体系，严厉打击违法犯罪，切实维护群众合法权益，全市社会治安形势稳定。

（六）改善民生福祉，市民生活水平稳步提高

居民收入增加。完善城乡统筹的就业机制，全市新增城镇就业12.8万人。积极创建国家级创业型城市，扶持自主创业1万人、带动就业6.45万人。亚太经合组织技能开发促进中心落户无锡，我市成为国家级高技能人才公共实训基地。企业职工最低月工资标准由850元提高到960元，全市企业退休人员月人均养老金增加到1432元，其中市区增加到1479元。推进物业、资金、劳务等农村富民合作，累计组建各类合作组织1546家，参加农户总数达55万户。城镇居民人均可支配收入27905元，农民人均纯收入13890元。社会保障统筹推进。继续扩大社会保险覆盖面，全市企业养老、基本医疗、失业保险参保人数分别增加到203.9万人、236.5万人、156.2万人。市区在全国率先推行城乡一体的居民养老保险和医疗保险制度，整合工作全面完成。城乡低保标准全面并轨，市区月低保标准提高至420元，共发放低保金1.6亿元。实施临时救助3.5万人次，发放各项慈善救助金1.67亿元。实事项目惠及群众。30项为民办实事项目全面完成。全市老新村整治改造和老住宅特修近177万平方米，其中市区近110万平方米。全市新开工经济适用房9900多套，其中市区7600多套。全市享受廉租房政策居民5233户，其中市区3800多户。市区竣工拆迁安置房773万平方米。全市新增居民燃气用户12.5万户，改造铺设乡镇自来水管道202千米。全市各类养老机构床位发展到2.1万张。为13515名残疾人提供免费体检服务，为全市所有贫困家庭残疾儿童提供免费康复训练服务。对口援建汉旺镇、清平乡任务圆满完成，累计完成总投资16.4亿元。

2010年市政府全面完成新一轮机构改革，进一步下放管理权限，深化行政审批制度改革，努力提高行政服务能力。加强廉政建设，推进权力阳光运行，确保各级行政机关依照法定权限和程序行使职权、履行职责。认真贯彻市人大及其常委会的各项决议，扎实办理人大代表的建议、批评、意见和政协委员的提案及建议案，努力提高政府工作实效。审计、史志、档案、侨务、对台、人武、民族宗教、防震减灾等工作取得新成绩，妇女、儿童、老龄、残疾人等事业有了新发展。

各位代表，2010年各项目标任务的顺利完成，标志着“十一五”发展圆满收官。五年来，无锡综合实力大幅提升，城乡面貌日新月异，人民生活显著改善，社会事业全面发展。与“十五”期末相比，全市地区生产总值翻番有余，人均地区生产总值突破9万元，财政总收入由421.8亿元增加到1579.9亿元，一般预算收入由181.7亿元增加到511.9亿元，无锡的发展跨上了新的更高平台，全面建成了小康社会，现代化建设取得了重大突破，一座充满活力、独具特色的江南名城以崭新形象屹立于太湖之滨。

这五年，我们坚持以转型发展为战略目标，全力推动发展方式根本转变。实施先进制造业和现代服务业“双轮驱动”战略，积极培育战略性新兴产业，大力发展以科技创新为核心的创新型经济，经济结构调整取得重大进展。高新技术产业增加值占规模以上工业增加值比重、服务业增加值占地区生产总值比重分别比“十五”期末提高12.3个百分点和5个百分点。集成电路制造技术和能力、光伏太阳能制造技术和产值跃居全国城市首位，成为惟一的国家传感网创新示范区和首批国家海外高层次人才创新创业基地。

这五年，我们坚持以统筹协调为基本方法，全力推进城市现代化和城乡一体化。着眼构筑“七区一体、一体两翼”发展格局，以城乡规划全覆盖为先导，以空间布局优化为基础，以公共设施建设为重点，以制度接轨为突破，大力推进区域城市化进程，多中心城市格局初步成型，交通枢纽地位进一步巩固，城市集聚辐射能力显著提升。五年新增一级公路551千米，新改建农村公路2006千米，市区新建及改拓建城市道路917千米，无锡机场成为国家一类口岸，无锡江阴港跨入“亿吨大港”行列，无锡成为国家历史文化名城。

这五年，我们坚持以环保优先为重要方针，全力开创生态文明新境界。把环境保护摆上经济社会发展先导位置，积极处理好加快发展和优化生态的关系，更加突出源头控制、过程控制、全程控制，逐步走上经济社会与人口资源环境相协调的发展新路。主要污染物排放量五年累计削减32%以上，单位地区生产总值能耗下降20%以上，均超额完成“十一五”目标任务。蠡湖水环境综合治理荣获全国人居环境范例奖，无锡建成全国首个生态城市群和全省惟一的国家森林城市，荣获国际花园城市殊荣。

这五年，我们坚持以改革创新为动力之源，全力提升体制机制竞争优势。围绕形成有利于科学发展的体制机制，全面深化经济、社会、行政管理体制改革，全力突破重点领域和关键环节，经济社会发展动能有效增强。民营经济占全市经济比重提高到63.5%，五年新增境内外上市企业33家，累计有75家世界五百强企业在锡投资发展，内外源经济协调发展格局基本形成。社会事业领域实现政事分开、管办分离，行政执行能力进一步提高。

这五年，我们坚持以改善人民生活为根本宗旨，全力构建幸福安康的和谐社会。把提高发展的普惠性作为工作着力点，扩大社会就业，提高居民收入，完善社会保障，加强公共服务，强化社会管理，力求让更多人共享改革发展成果。五年累计新增城镇就业58.7万人，城镇居民人均可支配收入和农民人均纯收入年均增长11.8%和11.7%，均超过人均地区生产总值增长速度。企业退休人员养老金连续五年增加，全市养老和医疗参保人数五年累计增加75.9万人和85.6万人。市区基本建立城乡一体的居民养老和医疗保障体系、社区卫生服务体系、公共文化和体育服务体系、公共交通服务体系。

各位代表，“十一五”发展的成就来之不易、令人鼓舞，发展的经验弥足珍贵、影响深远。经济社会发展取得的每一个成就、城市发生的每一处变化、百姓得到的每一点实惠，无不凝聚着全市人民的心血与汗水，无不饱含着方方面面的关心与支持，无不见证着600多万无锡市民的坚韧与智慧。在此，我代表无锡市人民政府，向在各个领域和不同岗位上辛勤劳动、作出贡献的全体市民，向给予政府工作积极支持的人大代表和政协委员，向各民主党派、工商联、各人民团体和各界人士，向中央及省在锡单位，向驻锡人民解放军、武警官兵和公安干警，向所有参与、支持和关心无锡建设与发展的海内外朋友，表示衷心的感谢，并致以崇高的敬意！

“十二五”奋斗目标

进入“十二五”，国际、国内形势正发生深刻变化，我们必须善于利用重要战略机遇期，顺势而为，积极作为。从国际看，世界经济格局出现新变化，经济全球化纵深推进，科技创新孕育新突破，新一轮产业转移加快推进，这对无锡来说是难得的历史机遇；从国内看，我国工业化、信息化、城镇化、市场化、国际化深入发展，经济转型加快，市场需求潜力巨大，为无锡加快转变经济发展方式提供了新的契机；从区域看，随着地区高速铁路、高速公路等快速交通体系加快完善，长三角一体化进程加速，区域产业布局、社会结构、城市功能、居民生活方式正发生深刻变化，无锡作为区域中心城市完全可以拓展更大的发展空间；从自身实际看，无锡已进入工业化后期发展阶段，综合实力极大提升，经济结构日趋优化，创新资源快速集聚，城市对外辐射力和影响力显著提高，发展活力进一步增强，为未来经济社会发展再上新台阶奠定了良好的基础。

在看到各方面有利条件的同时，我们更要清醒认识到，无锡发展也面临着诸多矛盾和挑战。国际金融危机影响深远，不稳定因素和不确定因素仍然较多，围绕市场、资源、人才、技术等的国际竞争日趋激烈，对我市的影响将更为深刻。国内区域和城市竞相发展，无锡人口密集，自然资源匮乏，发展空间狭小，生态环境质量还不够稳定，区位优势和竞争优势面临挑战，争先进位乃至保位的压力很大。新兴产业和服务业发展还不够快，产业结构仍不尽合理，消费对经济增长的贡献份额还不高，尤其是科技、教育、人才等创新要素与无锡城市地位和发展需要相比还显不足，创新发展的能力仍需进一步提升。常住人口总量增长较快，基本公共服务和社会保障压力加大，居民收入分配差距较大，一些积累性、突发性的社会矛盾较为突出，改善民生和维护社会稳定任务艰巨。同时，政府自身建设面临新的课题，仍须进一步转变职能，提高效能，更好地服务于人民群众。我们必须时刻保持清醒头脑，不断增强忧患意识，科学把握发展规律，积极应对各种挑战，切实解决突出问题，努力实现又好又快发展，让全体无锡市民过上更加幸福的生活。

“十二五”时期是我市率先实现基本现代化的重要时期，也是加快转变发展方式、推进战略转型的关键阶段。《中共无锡市委关于制定无锡市国民经济和社会发展第十二个五年规划的建议》提出了今后五年无锡国民经济和社会发展的指导思想、主要目标与主要任务，市政府据此组织制定了《无锡市国民经济和社会发展第十二个五年规划纲要（草案）》（以下简称《纲要》），提请大会审议。

《纲要》提出：“十二五”期间，无锡要以率先实现基本现代化为目标，继续走在长江三角洲和全国城市发展的前列，努力提高城乡居民的幸福感和满意度，不断增强无锡综合实力、自主创新能力、国际竞争力和可持续发展能力，让无锡人民安居乐业、城乡共同繁荣、社会公平正义，推动科学发展、率先发展、转型发展和富民强市建设取得更大突破。

主要发展目标是：人均地区生产总值年均增长10%，一般预算收入年均增长12%；高新技术产业增加值占规模以上工业增加值比重达55%，服务业增加值占地区生产总值比重达49.5%以上；全社会研发费用占地区生产总值比重达3%以上；单位地区生产总值能耗和二氧化碳排放量分别比“十一五”期末降低20%，主要污染物排放量在2010年基础上削减20%；实现居民收入增长和经济发展同步，力争全市劳动者报酬和居民收入实现倍增。

根据以上基本定位和总体目标，“十二五”期间必须进一步明晰战略思路，牢牢把握工作主动权。推动战略转型，是“十二五”发展的主要任务。转型发展是实现可持续发展、攀登现代化高峰的必由之路。必须着眼全面实现战略转型，精心组织实施加快转型发展的重点工作，全力以赴转变发展方式、创新发展模式、完善增长动力，推动经济、社会、文化的全面发展。坚持在发展中促转型，在转型中谋发展，提高发展的全面性、协调性和可持续性，确立区域竞争新优势。实现普惠于民，是“十二五”发展的根本目的。民生连着民心，民心凝聚民力，保障和改善民生是政府工作的根本出发点与落脚点。必须更加注重发展的质量与效益，努力创造起点公平、机会均等、人人得益的发展环境，使公平和正义成为构建和谐社会的基本导向。坚持把富裕百姓放在首要位置，千方百计促进就业创业，合理调节收入分配，增加居民工资性、经营性、财产性收入。健全和完善覆盖广、标准高的社会保障体系，加快发展各项社会事业，推进基本公共服务均等化，加强社会管理创新，强化公共安全保障体系建设，促进人的全面发展和社会全面进步。坚持城市引领，是“十二五”发展的重要动力。城市是转型发展、优化发展、集约发展的物理空间，也是大容量市场、高效率经济组织和新兴产业的成长载体，区域城市化和城市现代化依然是推动“十二五”战略转型的强劲引擎。必须以超前理念引领城市建设，以优质项目完善城市功能，以精致手笔凸显城市个性，进一步增强中心城市综合功能，着力提升城市服务的专业化和高效化程度。着眼城乡统筹，以同一张发展蓝图、同一个政策体系、同一套制度安排来落实和推进全市域的经济、社会、文化和生态建设，集约布局，集中建设，实现功能聚合、整体协调，建设城乡一体、和谐共荣的现代都市。打造创新城市，是“十二五”发展的战略基点。改革创新是发展之魂，集聚人才是发展之本。必须将“人才引领、创新驱动”确立为经济社会发展的根本策略，全面推进发展理念、体制机制与增长动力创新，全力推动发展转型。加大重点领域和关键环节改革力度，全面推进综合配套改革，使改革创新成为经济社会发展的主要驱动力。坚持科技、教育和人力资源建设统筹推进，着力打造人力强市，全面增强集成创新和引进消化吸收再创新能力，大幅提高科技进步对经济增长的贡献率，创建国家创新型城市。建设生态文明，是“十二五”发展的目标追求。生态文明是科学发展的重要内涵。必须围绕生态城宜居城目标，立足于实现经济社会与人口资源环境相协调，大力推进低碳、绿色、可持续发展。着力加强源头治理和长效机制建设，积极构建生态产业体系、生态环境体系、生态制度体系和生态文化体系，努力化生态优势为发展优势，进一步彰显山水园林城市特色，再现江南水乡韵味。提升文化内涵，是“十二五”发展的价值取向。文化是城市的内在气质，文化竞争力决定城市竞争力。实现“十二五”发展目标，必须大力提升城市文明程度与市民文明素质，提升城市精神气质与文化内涵，以文化凝聚人心，以文化荟集力量。必须将文化融合于经济发展、科技创新、城市建设和社会进步全过程，大力激发全社会创新创造活力，把无锡建设成优雅、时尚、精致的魅力之城。

2011年工作任务

今年是“十二五”开局之年，也是加快转变发展方式的关键之年，做好今年工作将为无锡率先实现基本现代化奠定扎实基础。政府工作要深入贯彻落实科学发展观，正确把握形势变化新特点，以“推动科学发展率先发展、建设创新无锡幸福无锡”为主题，以加快转型发展为主线，以“科教兴市、人才强市、质量与知识产权立市”为主战略，加快推进经济结构调整，大力加强自主创新，切实抓好节能减排，不断深化改革开放，着力保障和改善民生，努力保持经济平稳较快发展与社会和谐稳定。

综合考虑各方面因素，今年经济社会发展主要预期目标是：地区生产总值增长11.5%；万元地区生产总值能耗较2010年降低3.8%，主要污染物排放量较2010年削减4%；地方财政一般预算收入同口径增长12%；城镇居民人均可支配收入和农民人均纯收入均增长12%；城镇登记失业率控制在3.5%以内；居民消费价格指数控制在省定范围内。

根据上述目标和工作要求，必须重点抓好以下工作：

（一）保障和改善民生，提高社会建设水平

坚持以人为本、民生为先，大力发展各项社会事业，扎实办好11项为民办实事项目，使发展成果不断满足群众日益增长的物质文化需求。增加居民收入。继续实施积极的就业政策，多渠道增加就业岗位，做好重点人群就业工作，保障劳动者权益，构建和谐劳动关系。新增城镇就业10万人。健全面向全体劳动者的职业培训和技能人才培养体系，构建扶持创业体系，完善城乡一体的人力资源服务体系。规范分配秩序，调整企业最低工资标准和工资指导线，健全职工工资集体协商制度，做好企业退休人员养老金调整工作。拓展投资渠道，增加居民经营性和财产性收入，实现城乡居民收入较快增长。提升保障水平。全面推行城乡一体的养老保险和医疗保险制度，企业职工养老保险扩面10万人，适龄居民养老保险参保覆盖率达到96%；企业职工基本医疗保险扩面4万人，居民医疗保险覆盖率达到99%。完善以城乡居民最低生活保障和“五保”供养制度为基础的社会救助体系，推进普惠均等的社会福利体系建设。新增养老床位3000张、居家养老服务机构300家。加快建设南山慈善家园，建成市儿童福利院。完善住房保障体系，市区享受廉租房政策居民4200户以上，新增公共租赁住房、人才公寓1万套以上，建成交付经济适用房60万平方米。完成中心城区危旧房改造30万平方米，老新村整治改造90万平方米。促进社会进步。实施教育优先战略，创建全国义务教育优质均衡改革发展示范区。加快完善学前教育体系，对本市户籍贫困家庭的适龄幼儿免收保育教育费。推进高等教育重点项目建设。深化教育体制改革，鼓励社会力量出资、捐资办学。深入推进医药卫生体制改革，全面实施基本药物制度，整合公共医疗卫生资源，加强城乡公共卫生服务体系建设，全面深化公立医院改革。人均基本公共卫生服务经费提高到25元。完成市三院扩建和市中医院易地新建工程。完善公共文化服务体系，创建国家历史文化名城群，推动文化产业繁荣发展。加快八大博览园、五大历史文化街区及阖闾城遗址公园建设。完善全民健身服务体系，加强竞技人才培养，推进体育产业发展。办好第二十七届亚洲国际邮展、环太湖国际公路自行车赛、第二届中国湿地文化节暨亚洲湿地论坛等活动。

（二）增强内生增长动力，促进经济平稳健康运行

落实中央宏观调控政策措施，充分挖掘内需巨大潜力，不断提升开放型经济水平，积极完善消费投资出口协调拉动经济增长格局。优化内需结构。改善居民消费条件，积极引导各类业态错位布点，推进农贸市场标准化升级，吸引规模型内外资服务企业在锡建立区域总部，完善城乡商贸流通体系。社会消费品零售总额增长16%。加强市场供应统筹协调，综合运用多种手段调控价格水平，努力保持市场供应和价格总水平基本稳定。着力优化政府投资结构，加强政府性债务管理。积极鼓励和引导民间投资，不断提高投资质量和效益。全力推进和落实160个市级重点项目。全社会固定资产投资增长15%。深化经济改革。完善国有资产经营管理体制，健全非经营性资产、资源资产监管运行机制。支持企业跨区域、跨所有制重组。深化财税体制改革，规范预算外资金管理。推动企业资本经营，支持企业发行债券、中期票据，新增境内外上市企业12家，力争直接融资200亿元。提升国际化水平。进一步扩大利用外资规模，积极优化利用外资结构，不断提升引资质量和水平。鼓励企业加强出口品牌和营销网络建设，稳步提高一般贸易出口比重，重点扶持高新技术产品出口和服务外包发展。鼓励资源性产品进口，加快引进先进技术和设备。进出口总额增长8%。加强境外投资分类指导，鼓励有实力的企业组团“走出去”。加快开发区转型升级，提升产业集聚度和竞争力，增强综合服务功能。继续发展一批国际友好城市。

（三）推进经济结构优化升级，提升产业核心竞争力

坚持把经济结构战略性调整作为推动发展方式转变的主攻方向，加快构建以高新技术产业为主导、服务经济为主体、先进制造业为支撑、现代农业为基础的现代产业体系。强化服务业发展特色。开展“服务业超越促进年”活动，突出发展软件服务外包、金融、物流、会展等现代服务业，加快发展总部经济，推进21个城市综合体和23个现代服务业集聚区建设。服务业增加值占地区生产总值比重提高到44%。加快太湖新城中央商务区建设，引导国内外各类金融机构集聚发展。丰富旅游业态，培育旅游品牌，强化要素整合，提升旅游业国际化程度。合理调控房地产供应总量和结构，保持房地产市场平稳健康发展。放大新兴产业先发效应。创优新兴产业培育环境，落实扶持政策，重点建设25个新兴产业特色专业园区，力争战略性新兴产业营销收入超5000亿元。全力建好国家传感网创新示范区，实施感知电力、感知环保等应用示范工程。高起点建设一批生物产业中试平台，培育生物医药龙头企业。完善创意设计、动漫游戏产业链，推进无锡（国家）数字电影产业园、无锡数字动漫创业服务中心建设。加快发展信息服务产业，壮大软件骨干企业。创建国家云计算服务创新发展试点城市。增创制造业竞争优势。重点发展以自主品牌和自主技术为主的电子信息、高端装备等优势主导产业，促进信息化和工业化深度融合，加快支柱产业向集约化、高端化发展。高新技术产业增加值占规模以上工业增加值比重超过47.5%。推广卓越绩效管理模式，鼓励企业将品牌建设与科技创新、管理创新、营销创新结合起来，创建知名品牌，支持企业参与国际标准、国家标准和行业标准制订。提升现代高效农业规模水平。建设无锡太湖生物农业谷，培育生物育种、转基因生物反应器等生物农业新产业。做大做强一批农业龙头企业和农业品牌，建成一批上规模的优质稻米和设施化蔬菜生产基地，提高农产品自给率。强化农产品质量安全监管和重大动物疫病防控，确保农产品消费安全。

（四）加快城乡一体化发展，打造区域性中心城市

坚持中心城市带动战略，大力提升中心城市辐射能级和城乡集约建设水平，全面推进城乡一体化。优化空间布局。推进市域主体功能分区战略，明确发展导向，调控开发强度，促进均衡发展。着力形成以市区为核心、江阴宜兴城区为两翼、若干新城镇为支撑的城镇空间格局。严格落实基本农田保护制度，统筹安排各类建设用地，提高土地集约利用水平。深入推进万顷良田建设，实施土地整理4400公顷。突出新城建设。加快中心城市南进步伐，推进老城区有机更新。高起点高标准建设太湖新城，建成无锡大剧院、中央商务区一期、太湖国际博览中心二期、金桥小学等功能性项目。基本形成锡东新城核心区路网体系和交通枢纽功能，稳步推进蠡湖新城功能开发，大力提高科技新城（新区）国际竞争力，强化惠山新城产业特色，完善江阴临港新城综合功能，提升宜兴环科新城集聚水平。构建综合交通体系。拓展无锡机场客货运航线，启动二期改扩建工程，全年进出港旅客和货邮吞吐量分别达到300万人次和7万吨。加快地铁1号线建设，19个地下车站完成主体结构，盾构区间施工完成工作量一半以上。全面开工建设地铁2号线，完成地铁3、4号线建设规划编制和报批。建成沪宁城际高铁无锡站综合交通枢纽、京沪高铁无锡站。完成太湖大道节点改造，市区新建及改拓建城市道路260千米。加快锡澄运河改造整治工程，全面对接惠澄大道——疏港路等连接市区与江阴的重大交通基础设施，推进跨太湖通道建设，有效整合锡澄、锡宜地区生产力布局。继续优化公交线网结构，增加公交运力，完善场站设施，改善公交服务。推进现代化新农村建设。深入开展“幸福镇村”争创活动，加快农村流通网络建设，继续实施十大强农惠农工程。加快盘活农村各类资源，壮大村级实力，开辟农民增收新渠道。深入开展农村环境连片整治，有效控制农村生活污水和农业面源污染。统筹规划建设农村基础设施和公共服务设施，改造农村道路150千米，改造农村危旧桥梁150座。

（五）发挥科技人才支撑引领作用，创建国家创新型城市

坚持把人才作为科学发展的第一资源，深入推进科技创新创业，突出企业创新主体作用，促进科技人才资源高效配置和综合利用，加快建设创新型经济领军城市和科技创业家摇篮城市。加大招才引智力度。开展“人才特区建设促进年”活动，继续实施 “无锡千人计划”等系列海外引才计划，完善人才公共服务体系，加快国家海外高层次人才创新创业基地建设，壮大专业技术人才和高技能人才队伍。推进国有企业、民营企业与科技创新创业企业开展多方面合作。推动企业与国内外高校、科研院所进行战略合作，吸引跨国公司、科研院所来锡设立研发中心、分支机构，有效发挥在锡高校和科研院所作用。完善区域创新体系。支持有条件的企业加快研发机构建设，加强创新载体和平台建设。引导社会资金成立或参与各类创业投资基金，形成科技金融集团化服务优势。继续鼓励商业银行试办科技支行、科技信贷专营部门，发展以企业信用为基础的股权质押、知识产权质押等信贷产品，建设科技型中小企业信用体系试验区。全面提高知识产权创造、运用、保护和管理能力。全社会研发费用占地区生产总值比重超过2.6%，其中企业研发费用占全社会研发费用比重超过90%。加快城市信息化建设。在“无线无锡”工程基础上，完善网络基础设施，提高网络服务水平和业务承载能力。推进跨部门业务协同系统建设，加快信息资源开发利用和共享交换进程。推进社区服务管理信息化，创新社会管理手段。推进社会事业信息化，逐步实现资源数字化、服务网络化。

（六）加强环境保护和生态建设，夯实可持续发展基础

围绕生态文明先驱城市建设目标，深入实施环境综合治理，积极探索以绿色、低碳为特征的发展新路，全面增强可持续发展能力。高起点建设低碳城市。严格控制、逐步降低燃煤总量，积极发展和利用光伏等新能源，重点实施光伏太阳能推广应用示范工程和可再生能源建筑应用示范工程。开发和推广资源节约、替代和循环利用技术，实施一批重点节能和循环经济项目。启动中瑞低碳生态城建设。高标准实施环境治理。扎实推进太湖（蠡湖）新城流域、直湖港流域、望虞河两岸、太湖西部沿岸等重点区域水环境治理，继续实施梅梁湖生态清淤等项目，完成走马塘拓浚延伸河道工程。基本建成“排水用户全接管、污水管网全覆盖、污水处理厂全提标”的污水治理体系，城镇生活污水集中处理率达到95%。加大饮用水源地保护力度，集中式饮用水源地水质达标率稳定达到100%。强化自来水预处理和深度处理工艺，加快中远期保障优质安全供水工程建设，确保居民用水安全。加大工业废气、机动车尾气和建筑工地扬尘防治，加强大气自动监测体系建设。扩大建成区机动车24小时禁鸣范围。建立和完善城镇生活垃圾收集和处理网络，加大土壤污染、固体废弃物和持久性有机污染物防治力度，城市生活垃圾无害化处理率100%。高水平营造绿色家园。加快城乡绿道网建设，发展垂直绿化、屋顶绿化，建成金城湾公园、长广溪湿地二期、贡湖湾湿地和雪浪山生态景观公园。完成造林绿化3300公顷以上，全市林木覆盖率达到25%，建成区绿化覆盖率达到43.8%，环境质量综合指数稳定达到90。

（七）提高依法行政能力，建设法治政府服务政府

适应经济社会转型发展需要，全面履行政府职能，切实加强政府自身建设。坚持依法行政。开展重点领域专项执法检查，规范行政执法工作。完善听证制度和重大决策的调查研究、专家论证、群众评议制度，优化政府决策规则和程序。健全政府信息公开和新闻发布制度，推进行政权力网上公开运行，加强行政权力运行监督。自觉接受人大法律监督、工作监督和政协民主监督，主动接受群众监督和舆论监督。加强社会管理。完成街道行政管理体制改革，城乡社区扁平化管理实现全覆盖。深化人口管理体制创新，建立健全人口发展调控机制。实施新型居住证管理制度，逐步实现常住人口同城待遇。不断强化城市管理，全面提高数字化、精细化管理水平。扎实开展法制宣传教育，提升市民法律素质。强化市场监管，进一步加强农产品安全体系和肉类蔬菜流通追溯体系建设，基本建成食品药品检验检测中心，努力保障食品药品安全。加大公共安全投入，建立健全自然灾害、事故灾难、公共卫生事件、社会安全事件预防预警和应急处置体系。强化基础能力建设，提升灾害预防能力，全面提高防震减灾综合水平。进一步落实企业安全生产主体责任，确保安全生产形势稳定。强化维稳机制建设，提升社会矛盾疏解能力，深化社会治安防控体系建设，加强社会管理创新，提高公正廉洁执法水平，促进社会和谐稳定。争创全国文明城市。争创全国双拥模范城“六连冠”。提高服务效能。进一步削减行政许可事项，简化行政许可环节，为企业和市民提供优质便捷的政务服务。积极推进行政事业性收费改革，清理、削减和规范行政收费行为。加强对经济社会发展重要决策部署的监督检查，确保各项政策措施全面落实。强化领导干部经济责任审计，完善权力运行制约监督机制。加强政府廉政建设，健全预防和惩治腐败体系，严肃查处违法违纪案件和严重损害群众利益的案件。加强政府公务员队伍教育、培训和管理，提高服务发展、服务基层、服务群众的能力和水平。

各位代表，我们正站在一个新的历史时点，推动科学发展率先发展、建设创新无锡幸福无锡是全市人民的共同追求。让我们紧密团结在以胡锦涛同志为总书记的党中央周围，在江苏省委省政府和无锡市委的坚强领导下，开拓创新，锐意进取，求真务实，团结拼搏，为率先实现基本现代化而努力奋斗！
